# Multimodal Integration of Gait Dysfunction, Amyloid PET, and Plasma Biomarkers for Differentiating Etiological Subtypes in Mild Cognitive Impairment

**DOI:** 10.1002/cns.70949

**Published:** 2026-06-05

**Authors:** Jiaonan Wu, Fang Tang, Xinyi Lv, Yiwei Wang, Feng Gao, Yong Shen, Zhaozhao Cheng, Jiong Shi

**Affiliations:** ^1^ Department of Neurology, the First Affiliated Hospital of USTC, Division of Life Sciences and Medicine University of Science and Technology of China Hefei China; ^2^ Graduate School Bengbu Medical University Bengbu China; ^3^ Neurodegenerative Disorder Research Center, Division of Life Sciences and Medicine University of Science and Technology of China Hefei China

**Keywords:** amyloid PET, dual‐task gait, gait analysis, mild cognitive impairment, plasma biomarkers

## Abstract

**Objective:**

To investigate gait characteristics and plasma biomarkers in individuals with mild cognitive impairment (MCI) stratified by amyloid‐β (Aβ) positivity on positron emission tomography (PET), and to evaluate the predictive value for Alzheimer's disease (AD)–related MCI.

**Methods:**

A total of 168 participants were enrolled, including 50 amyloid PET‐negative MCI (MCI‐), 51 amyloid PET‐positive MCI (MCI+), and 61 cognitively normal (CN) individuals. Gait assessments were conducted using a multi‐sensor motion analysis system during dual‐task paradigms (cognitive load superimposed on locomotion). Plasma samples were analyzed for neurofilament light chain (NfL), glial fibrillary acidic protein (GFAP), and phosphorylated tau at threonine 217 (p‐tau217) via ultra‐sensitive immunoassays.

**Results:**

Gait analyses identified 125 features, with 69 distinguishing MCI+ from CN and 36 differentiating MCI+ from MCI‐. Receiver operating characteristic (ROC) analyses showed that dual‐task gait under the countdown and animal‐naming conditions (gait‐countdown [GCD] and gait‐animal naming [GAN]) discriminated MCI+ from CN with an AUC of 0.850. The combined models integrating GCD and GAN with plasma GFAP or p‐tau217 yielded AUCs of 0.919 and 0.951, respectively. Similarly, GCD&GAN features demonstrated an AUC of 0.862 for distinguishing MCI+ from MCI−, with GFAP (AUC = 0.933) and p‐tau217 (AUC = 0.986) enhancing predictive performance.

**Conclusion:**

This study provides evidence that dual‐task gait assessments, when combined with plasma biomarkers such as GFAP and p‐tau217, may improve the discriminatory power for identifying AD‐related MCI. The integration of biomechanical and molecular markers holds promise for advancing early detection strategies and therapeutic monitoring in AD.

## Graphical abstract

This study demonstrates that dual‐task gait assessments combined with plasma biomarkers (GFAP and p‐tau217) significantly improve the discrimination of amyloid‐positive mild cognitive impairment (MCI+) from both cognitively normal individuals and amyloid‐negative MCI. The integrated models achieved AUCs of 0.951 and 0.986, respectively, highlighting the potential of multimodal approaches for early detection of Alzheimer's disease‐related cognitive decline.

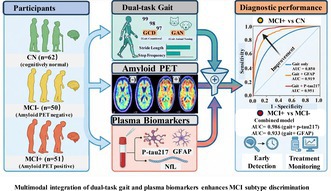

**Abbreviations**
AD: Alzheimer's disease
AN: Animal Naming
APOE: Apolipoprotein E
AR: accuracy rate
BMI: body mass index
CANDI: China Aging and Neuro‐degenerative Initiative
CD: Countdown
CDR: Clinical Dementia Rating
CN: cognitively normal
CSF: Cerebrospinal Fluid
DTC: dual‐task cost
GAN: gait‐animal naming
GCD: gait‐countdown
MCI: mild cognitive impairment
MCI: amyloid PET‐negative MCI
MCI+: amyloid PET‐positive MCI
MMSE: Mini‐Mental State Examination
MoCA: Montreal Cognitive Assessment
PET: positron emission tomography
ROC: Receiver operating characteristic
SAN: standing AN
SCD: standing CD
USTC: University of Science and Technology of China

## 1 Introduction

Mild cognitive impairment (MCI) represents a transition state between normal aging and dementia. While MCI patients experience mild cognitive decline, their ability to perform daily activities generally remains unimpaired [1]. Epidemiological evidence indicates that approximately 10% to 15% of MCI patients progress to Alzheimer's disease (AD) on an annual basis [1].

The defining pathological hallmarks are the formation of β‐amyloid plaques and tau protein neurofibrillary tangles. These neuropathological alterations emerge at an early stage of AD [2], potentially preceding the onset of MCI, and gradually intensify as the disease progresses [3]. The cumulative effect of these changes culminates in neuronal damage and cell death, ultimately leading to severe cognitive impairment [4].

With the recent evolution of neuroimaging modalities, particularly the advent of amyloid positron emission tomography (PET), researchers have gained the ability to categorize MCI patients with greater precision. MCI can now be stratified into two primary subgroups: AD‐related MCI (MCI+) and non‐AD MCI (MCI‐) [5]. MCI+ patients exhibit significant β‐amyloid deposition on amyloid PET scans, positioning them at an elevated risk of progressing to AD [5, 6, 7, 8, 9]. Conversely, MCI‐ patients lack β‐amyloid deposition, with their cognitive decline potentially attributable to alternative etiologies, such as vascular lesions or other neurodegenerative diseases [6, 8, 10]. The emergence of anti‐amyloid therapies has heightened the urgency of distinguishing between MCI+ and MCI‐ patients to enable targeted treatment strategies [8].

Longitudinal studies have demonstrated that MCI patients with positive amyloid PET scans exhibit a higher conversion rate to AD compared to those with negative scans [11]. Early identification of MCI+ patients is of great clinical importance, as it enables the timely initiation of disease‐modifying therapies, which have the potential to slow disease progression [12]. Additionally, early diagnosis provides patients and their families with the opportunity to access necessary support services and resources, facilitating better preparation for future challenges [13, 14]. Despite its diagnostic utility, amyloid PET scans are associated with significant limitations, including high costs and the use of radioactive tracers, which impede their widespread application in population‐based screening [15]. Furthermore, methodological variability in imaging analysis and tracer types complicates cross‐study comparisons [16].

Gait analysis, a non‐invasive, accessible, and cost‐effective assessment method, holds promise as a tool for the early detection of MCI. Single‐task gait analysis, which evaluates walking patterns under unperturbed conditions, has revealed characteristic abnormalities in MCI patients, including shortened step length and reduced gait speed [17, 18]. Dual‐task gait analysis, which incorporates concurrent cognitive tasks (such as counting, naming, etc.) during walking, offers additional insights into the impact of cognitive load on locomotion. MCI patients typically exhibit greater gait variability and more disrupted rhythmicity under dual‐task conditions, features that effectively discriminate them from cognitively normal elderly individuals [19, 20, 21, 22].

Advancements in ultrasensitive assay technologies have enabled the quantification of plasma‐based biomarkers as alternatives to cerebrospinal fluid (CSF) biomarkers. Among these, phosphorylated tau at residue 217 (p‐tau217) has emerged as a highly robust biomarker [23, 24], demonstrating strong correlations with cerebral amyloid‐β (Aβ) deposition and tau pathology [25, 26]. Plasma neurofilament light chain (NFL) serves as a non‐specific indicator of axonal injury [27, 28], with longitudinal analyses demonstrating its progressive elevation across the AD continuum, reflecting accelerated brain atrophy and predicting future cognitive decline [29]. Glial fibrillary acidic protein (GFAP), a marker of astrocytic activation and neuroinflammation secondary to neuronal damage [30], exhibits elevated plasma levels prior to the appearance of amyloid PET positivity [31]. Collectively, these plasma biomarkers offer a more accessible and less invasive approach to AD diagnosis and disease monitoring compared to traditional CSF or PET‐based methods.

The primary objective of this study was to evaluate the diagnostic utility of gait analysis in the identification of MCI patients, with a particular focus on MCI+ individuals, to facilitate early intervention. Additionally, we sought to systematically explore the diagnostic potential of recently investigated plasma AD biomarkers and to determine whether the integration of multimodal gait parameters with plasma biomarkers could enhance the sensitivity of disease progression monitoring and risk stratification in MCI+ patients.

## 2 Methods

### 2.1 Participants

The China Aging and Neuro‐degenerative Initiative (CANDI) cohort is a longitudinal study initiated in 2018. It enrolls patients with CN, MCI, and dementia [32] with a comprehensive evaluation of cognitive function, MRI images, multi‐tracer PET scans, and biospecimens. All subjects in this study were from the CANDI cohort.

A total of 168 participants who were willing to have gait analysis from November 30, 2022, to August 15, 2024, were included in this study. Based on Clinical Dementia Rating (CDR) scores, participants were categorized into CN and MCI groups. CDR = 0 was defined as CN, and CDR = 0.5 as MCI. These 101 were further subdivided based on amyloid PET results into amyloid‐positive MCI (*n* = 51) and amyloid‐negative MCI (*n* = 50). Sixty‐seven CN individuals were enrolled in the study. After excluding six amyloid‐positive cases, a total of 61 CN individuals were included.

Inclusion criteria for MCI patients: (1) Diagnosed according to the 2018 NIA‐AA research framework guidelines with a CDR of 0.5 [5]. (2) Ability to walk independently and perform basic calculations. (3) No history of serious falls within the 12 months prior to participating in the study. (4) Sufficient visual and hearing ability to appropriately respond to verbal instructions. (5) At least 5 years of education.

Exclusion criteria for MCI patients: (1) Unwillingness or inability to complete all tests. (2) Cognitive impairment caused by other degenerative diseases or encephalitis, epilepsy, brain trauma, metabolic encephalopathy, drug and substance abuse, or psychiatric disorders, etc.

Inclusion criteria for CN: (1) **CDR = 0**. (2) Same as MCI patients for criteria 2, 3, 4, and 5. Exclusion criteria were the same as those for MCI patients.

The protocol was approved by the First Affiliated Hospital of USTC's Ethics Committee (IRB#2019KY‐26, new revision supplement: 2023KY‐117). Written informed consent was obtained from all participants.

### 2.2 Neuropsychological Assessment

In this study, assessments were conducted by two experienced evaluators, including the Activity of Daily Living (ADL), CDR, and Mini‐Mental State Examination (MMSE). The MMSE was used to provide an overall assessment of cognitive function. Permission has been obtained to use the MMSE and Montreal Cognitive Assessment (MoCA). Evaluators conducted semi‐structured interviews with participants and reliable informants to assess CDR.

Two cognitive tasks were performed while standing for 60 s: (1) Countdown (CD): Participants are required to count down from 100 to 1 (e.g., 99, 98, 97.); (2) Animal Naming (AN): Participants are asked to name animals as many as possible. Both tasks required participants to respond verbally. To assess cognitive performance, the accuracy rate (AR) was calculated for each cognitive task, where AR = the number of correct responses/total number of responses x 100%.

### 2.3 Apolipoprotein E (APOE) Genotype

The apolipoprotein E (APOE) genotype was determined following a previously described protocol [32], using PCR amplification and HhaI digestion (NEB, R0139S). APOE utilizes genotype data from two SNPs to determine ε2, ε3, and ε4 alleles.

### 2.4 Fluid Biomarker Collection and Measurements

Blood samples were collected after overnight fasting and centrifuged for 2 h to remove cell debris. Aliquots of plasma were stored at −80°C until analysis. Measurements of plasma Ab42, Ab40, and p‐tau 217 were performed using Simoa kits (Quanterix, 103,714, 101,195) on an HD‐X analyzer (Quanterix, Billerica, USA) as per the manufacturer's instructions (Quanterix, 103,714, 101,195). Serum glial fibrillary acidic protein (GFAP) and neurofilament light chain (NFL) were also measured using Simoa kits (Quanterix, 103,520).

### 2.5 18F‐Flobetapir (AV‐45) PET Scans

Amyloid PET imaging was completed by 168 participants using a PET/computed tomography (CT) scanner (BioGraph 16HR; Siemens Healthcare, Erlangen, Germany). The scan details were documented in the CANDI cohort [32, 33], with results visually interpreted by two nuclear medicine specialists [33, 34].

### 2.6 Single‐Task Gait Assessment

The gait parameter assessment was performed on the ReadyGo quantitative movement assessment system (Beijing Zhong Ke Institute of Ruixi Information Technology Co. Ltd.). The ReadyGo system employed advanced artificial intelligence technologies such as depth visual sensing, camera‐based capture for 3D human body reconstruction, and dynamic tracking of skeletal points for three‐dimensional motion capture analysis. Participants did not need to wear sensors with this device, as it captured 3D motion and located skeletal points using a set of cameras. Furthermore, the gait testing did not require a specific room setup; it only required an obstacle‐free area of 1 × 5 square meters in front of the cameras. In the single‐task gait test, participants walked at a natural pace along a 3‐m walkway.

### 2.7 Dual‐Task Gait Assessment

Our study includes two dual‐task paradigms: (1) gait‐countdown (GCD): Participants were required to count backwards from 100 while performing the gait task. (2) gait‐animal naming (GAN): During dual‐task conditions, they were asked to name as many animals as possible while walking.

Dual‐task cost (DTC) was used to assess motor‐cognitive interaction, reflecting changes in gait parameters under dual‐task conditions. The influence of cognitive tasks on gait performance is quantified as a percentage using the formula: DTC = ([single‐task parameter‐ dual‐task parameter]/single‐task parameter) × 100% [35].

### 2.8 Statistical Analyses

Statistical analyses included *t*‐tests, chi‐square tests, Mann‐Whitney U tests, and correlation analyses (Pearson/Spearman), depending on variable distribution. Correlations between plasma biomarkers and gait parameters were primarily assessed using Pearson's correlation when variables were approximately normally distributed; Spearman's rank correlation was additionally performed as a sensitivity analysis for non‐normally distributed variables. To account for multiple testing in the correlation analyses, false discovery rate (FDR) correction was performed using the Benjamini‐Hochberg method. Binary logistic regression examined demographic and clinical impacts on gait parameters in MCI patients. ROC curve analysis assessed the gait analysis's diagnostic performance. DeLong tests were used to compare the AUCs of biomarker‐only models with those of the corresponding gait‐plus‐biomarker combined models. A significance level of *p* < 0.05 was applied.

All analyses were performed using IBM SPSS 27.0 (NY, USA) and GraphPad Prism 8.0 (GraphPad Software; La Jolla, USA).

## 3 Result

### 3.1 Participant Characteristics

The demographic and clinical characteristics of the MCI+, MCI‐, and the CN groups were summarized in Table 1, showing differences in age, body mass index (BMI), education, MMSE, ADL score, APOE4‐e4 carriers, plasma Aβ42, p‐tau217, NFL, and GFAP between the MCI+ and CN groups. There were significant differences in MMSE, ADL, APOE4 carriers, plasma Aβ42, Aβ40, p‐tau217, and GFAP between the MCI+ and MCI‐ groups.

**TABLE 1:** Baseline characteristics of enrolled participants.

Demographics	CN	MCI+	MCI‐	P1 (CN vs. MCI+)	P2 (MCI‐ vs. MCI+)
*n*	61	51	50		
Age (year), mean ± SD	59.72 ± 10.49	65.67 ± 7.74	63.91 ± 8.49	< 0.001	0.324
Gender (female), *n* (%)	35 (57.38)	28 (54.9)	26 (74.3)	0.793	0.068
Education (years), mean ± SD	11.67 ± 3.38	9.55 ± 3.49	9.43 ± 3.47	< 0.001	0.875
BMI, mean ± SD	24.35 ± 3.11	22.49 ± 2.76	23.60 ± 3.29	0.001	0.094
MMSE, mean ± SD	27.62 ± 1.71	20.82 ± 3.81	23.37 ± 3.15	< 0.001	0.002
ADL, mean ± SD	20.26 ± 0.95	23.35 ± 3.40	21.74 ± 2.61	< 0.001	0.02
APOE4‐e4carriers, *n* (%)	18 (30.00)	28 (57.14)	7 (20.00)	< 0.001	< 0.001
APOE genotype, *n* (%)				< 0.001	< 0.001
e2/e3	11 (18.33)	3 (6.12)	3 (8.57)		
e2/e4	3 (5.00)	4 (8.16)	2 (5.71)		
e3/e3	31 (51.67)	18 (36.73)	25 (71.43)		
e3/e4	11 (18.33)	20 (40.82)	2 (5.71)		
e4/e4	4 (6.67)	4 (8.16)	3 (8.57)		
Plasma Aβ42 (pg/mL), mean ± SD	10.85 ± 2.20	8.22 ± 2.27	10.62 ± 2.75	< 0.001	< 0.001
Plasma Aβ40 (pg/mL), mean ± SD	176.56 ± 30.32	166.37 ± 32.97	189.15 ± 41.39	0.125	0.014
Plasma *p*‐tau217 (pg/mL), mean ± SD	0.24 ± 0.18	0.76 ± 0.34	0.19 ± 0.09	< 0.001	< 0.001
Plasma NFL (pg/mL), mean ± SD	17.94 ± 20.06	27.05 ± 11.25	27.13 ± 25.19	0.006	0.986
Plasma GFAP (pg/mL), mean ± SD	118.45 ± 84.81	262.55 ± 138.93	150.78 ± 95.36	< 0.001	< 0.001

Abbreviations: Aβ40, amyloid‐beta 40; Aβ42, amyloid‐beta 42; ADL, activity of daily living; BMI, body mass index; CN, cognitively normal; GFAP, glial fibrillary acidic protein; MCI+, amyloid PET‐positive mild cognitive impairment; MCI‐, amyloid PET‐negative mild cognitive impairment; MMSE, Mini‐Mental State Examination; NFL, neurofilament light chain.

### 3.2 Differences in Gait Features Among the MCI+, MCI‐, and CN Groups

In single‐task gait analysis, 6 gait features differed between the CN and MCI+ groups. They are Gait‐Time, Gait‐Frequency_Left, Gait‐Stride Speed_Right, Gait‐Stride Speed_Left, Gait‐Swing Speed_Right, and Gait‐Turn Time (*p* < 0.05). Independent samples *t*‐test showed that only Gait‐Frequency_Right was significantly different between the MCI+ and MCI‐ groups (104.43 ± 11.42 vs. 109.89 ± 12.23, *p* = 0.042) (Table S1).

### 3.3 Differences in Dual‐Task Gait Features Among MCI+, MCI, and CN Groups

Comparison of dual‐task gait features between CN and MCI+ groups. In GCD, 17 gait features revealed differences (*p* < 0.05), with 10 features revealing significant differences (*p* < 0.001) (Table 2). In DTC‐GCD, 15 gait features displayed differences (*p* < 0.05), with 4 features displaying significant differences (*p* < 0.001) (Table 3). In GAN, 17 gait features revealed differences (*p* < 0.05), with 8 features revealing significant differences (*p* < 0.001) (Table S2). In DTC‐GAN, 14 gait features showed differences (*p* < 0.05), with 5 features showing significant differences (*p* < 0.001) (Table S3).

**TABLE 2:** Dual‐task GCD among MCI+, MCI‐, and CN groups.

Features	CN	MCI+	MCI‐	P1 (CN vs. MCI+)	P2 (MCI‐ vs. MCI+)
Test Time	50.32 ± 18.14	66.46 ± 25.16	54.46 ± 18.76	< 0.001	0.021
Standing Left	68.58 ± 1.76	69.89 ± 2.49	69.11 ± 2.89	0.005	0.227
Standing Right	68.54 ± 1.87	70.28 ± 2.71	69.00 ± 2.60	< 0.001	0.05
Swing Left	31.41 ± 1.76	30.10 ± 2.49	30.88 ± 2.89	0.005	0.227
Swing Right	32.34 ± 3.09	29.71 ± 2.71	30.99 ± 2.60	< 0.001	0.05
Bilateral Support Left	37.31 ± 3.13	39.85 ± 4.45	38.46 ± 4.75	0.003	0.209
Bilateral Support Right	37.27 ± 3.01	39.49 ± 4.39	38.13 ± 4.16	0.007	0.193
Stride Width	0.13 ± 0.02	0.13 ± 0.02	0.12 ± 0.03	0.576	0.441
Stride Left	1.13 ± 0.19	1.07 ± 0.17	1.07 ± 0.14	0.12	0.91
Stride Right	1.14 ± 0.21	1.09 ± 0.18	1.08 ± 0.15	0.17	0.862
Height Left	0.12 ± 0.02	0.12 ± 0.02	0.12 ± 0.02	0.86	0.637
Height Right	0.11 ± 0.02	0.11 ± 0.02	0.11 ± 0.02	0.696	0.437
Speed	0.86 ± 0.21	0.70 ± 0.22	0.81 ± 0.19	< 0.001	0.03
Frequency Left	97.52 ± 14.02	84.69 ± 17.26	93.38 ± 19.21	< 0.001	0.05
Frequency Right	96.25 ± 13.64	83.10 ± 17.46	93.17 ± 18.46	< 0.001	0.022
Stride Velocity Left	0.91 ± 0.21	0.75 ± 0.21	0.83 ± 0.20	< 0.001	0.102
Stride Velocity Right	0.93 ± 0.21	0.75 ± 0.21	0.84 ± 0.20	< 0.001	0.091
Swing Velocity Left	2.20 ± 0.41	1.86 ± 0.44	2.07 ± 0.44	< 0.001	0.042
Swing Velocity Right	2.22 ± 0.39	1.86 ± 0.44	2.05 ± 0.42	< 0.001	0.138
Turn Time	1.55 ± 0.59	1.92 ± 0.93	1.90 ± 0.80	0.032	0.934
Coordination	0.38 ± 8.50	1.02 ± 7.82	−1.42 ± 6.64	0.701	0.164
Stride Time Variance Left	7.70 ± 4.87	12.95 ± 9.77	7.70 ± 5.17	0.002	0.004
Stride Time Variance Right	7.80 ± 4.51	12.21 ± 9.61	8.65 ± 6.26	0.007	0.075
Frequency Variance Left	11.70 ± 9.91	9.12 ± 8.89	7.89 ± 6.74	0.186	0.52
Frequency Variance Right	10.17 ± 7.70	11.26 ± 9.46	9.22 ± 7.84	0.535	0.329

Abbreviations: CN, cognitively normal; GCD, gait‐countdown; MCI+, amyloid PET‐positive mild cognitive impairment; MCI‐, amyloid PET‐negative mild cognitive impairment.

**TABLE 3:** DTC‐ GCD among MCI+, MCI‐, and CN groups.

Features	CN	MCI+	MCI‐	P1 (CN vs. MCI+)	P2 (MCI‐ vs. MCI+)
Test Time	−0.24 ± 0.38	−0.53 ± 0.49	−0.28 ± 0.38	0.002	0.019
Standing Left	−0.02 ± 0.02	−0.03 ± 0.03	−0.03 ± 0.07	0.005	0.965
Standing Right	−0.02 ± 0.03	−0.04 ± 0.04	−0.02 ± 0.04	0.001	0.091
Swing Left	0.03 ± 0.05	0.07 ± 0.06	0.06 ± 0.10	0.006	0.609
Swing Right	−0.00 ± 0.17	0.08 ± 0.07	0.05 ± 0.08	0.004	0.087
Bilateral Support Left	−0.06 ± 0.08	−0.12 ± 0.11	−0.11 ± 0.13	0.002	0.51
Bilateral Support Right	−0.06 ± 0.08	−0.12 ± 0.11	−0.08 ± 0.11	0.003	0.183
Stride Width	−0.03 ± 0.09	−0.02 ± 0.11	0.02 ± 0.09	0.426	0.205
Stride Left	0.06 ± 0.07	0.08 ± 0.09	0.05 ± 0.08	0.253	0.114
Stride Right	0.06 ± 0.08	0.06 ± 0.11	0.06 ± 0.06	0.794	0.955
Height Left	−0.00 ± 0.20	0.07 ± 0.12	−0.00 ± 0.15	0.06	0.034
Height Right	0.06 ± 0.17	0.11 ± 0.13	−0.01 ± 0.12	0.121	< 0.001
Speed	0.16 ± 0.17	0.30 ± 0.18	0.21 ± 0.14	< 0.001	0.015
Frequency Left	0.11 ± 0.10	0.21 ± 0.13	0.16 ± 0.13	< 0.001	0.128
Frequency Right	0.13 ± 0.09	0.21 ± 0.13	0.16 ± 0.13	< 0.001	0.094
Stride Velocity Left	0.18 ± 0.12	0.27 ± 0.16	0.21 ± 0.15	0.003	0.078
Stride Velocity Right	0.17 ± 0.12	0.27 ± 0.16	0.21 ± 0.14	0.002	0.112
Swing Velocity Left	0.14 ± 0.11	0.24 ± 0.15	0.15 ± 0.15	< 0.001	0.015
Swing Velocity Right	0.15 ± 0.10	0.23 ± 0.13	0.18 ± 0.13	0.001	0.081
Turn Time	0.05 ± 0.35	−0.02 ± 0.40	0.01 ± 0.37	0.425	0.784
Coordination	0.55 ± 1.41	0.41 ± 1.17	1.13 ± 1.08	0.733	0.124
Stride Time Variance Left	−0.57 ± 1.39	−1.36 ± 1.66	−0.61 ± 1.12	0.014	0.027
Stride Time Variance Right	−0.80 ± 1.21	−0.98 ± 1.04	−1.17 ± 2.15	0.448	0.631
Frequency Variance Left	−0.91 ± 2.62	−0.67 ± 1.68	−0.39 ± 1.08	0.611	0.443
Frequency Variance Right	−0.76 ± 1.98	−3.07 ± 14.81	−1.45 ± 4.25	0.26	0.57

Abbreviations: CN, cognitively normal; DTC‐ GCD, dual‐task cost‐ gait countdown; MCI+, amyloid PET‐ positive mild cognitive impairment; MCI‐, amyloid PET‐ negative mild cognitive impairment.

#### 3.3.1 Comparison of Dual‐Task Gait Features Between MCI‐ Group and MCI+ Group

In GCD, there were 5 gait features that displayed significant differences (*p* < 0.05) (Table 2). In DTC‐GCD 6 gait features displayed differences (*p* < 0.05), with DTC‐ GCD_ Height‐ Right displaying a significant difference (*p* < 0.001) (Table 3). In GAN, 12 gait features revealed differences (Table S2). In DTC‐GAN, there were 12 gait features that revealed differences (*p* < 0.05) (Table S3).

### 3.4 Factors Independently Associated With Dual‐Task Gait Analysis in MCI+

Binary logistic regression analysis was performed on those gait features with significant differences (*p* < 0.001), adjusting for MMSE, ADL, age, gender, education level, and BMI. For MCI+ vs. CN, the results showed that DTC‐GCD_Frequency‐Right and DTC‐GAN_Gait Speed showed significant differences (Table 4). The differences between MCI+ and MCI‐ groups include DTC‐GCD_Height‐Right, DTC‐GAN_SwingSpeed‐Right, DTC‐GAN_Stance‐Right, DTC‐GAN_Stride‐Right, and DTC‐GAN_StrideSpeed‐Left (Table 4).

**TABLE 4:** Binary logistic regression showing factors independently associated with dual‐task gait analysis in MCI+ patients.

	B	SE	Wald	Sig.	OR (95% CI)
MCI+ vs. CN					
DTC‐ GCD_ Frequency‐ Right	19.806	7.411	7.141	0.008	3.996E+8 (196.333 ~ 8.134E+14)
DTC‐ GAN_ Gait Speed	7.162	3.019	5.625	0.018	1288.867 (3.468 ~ 479062.574)
MCI+ vs. MCI—					
DTC‐ GCD_ Height‐ Right	8.514	2.893	8.663	0.003	4983.897 (17.192 ~ 1,444,839)
DTC‐ GAN_ Stance‐ Right	−41.062	14.229	8.328	0.004	0.000 (0.000 ~ 0.000)
DTC‐ GAN_ Stride‐ Right	14.189	6.194	5.247	0.022	1452909.479 (7.754 ~ 2.722E+11)
DTC‐ GAN_ Stride Speed‐ Left	−30.268	10.508	8.298	0.004	0.000 (0.000 ~ 0.000)
DTC‐ GAN_ Swing Speed‐Right	28.996	10.347	7.852	0.005	3.914E+12 (694.359 ~ 2.514E+21)

*Note:* Multivariate logistic model predicting MCI+ after adjusting for MMSE, ADL, age, gender, education level, and BMI.

Abbreviations: B, beta coefficient; CI, confidence interval; DTC‐ GAN, Dual‐task cost‐ gait‐ animal naming; DTC‐ GCD, dual‐task cost‐ gait countdown; OR, odds ratio; SE, standard error; Sig, significance.

### 3.5 Correlation Between Plasma Biomarkers and Gait Parameters

**FIGURE 1:**
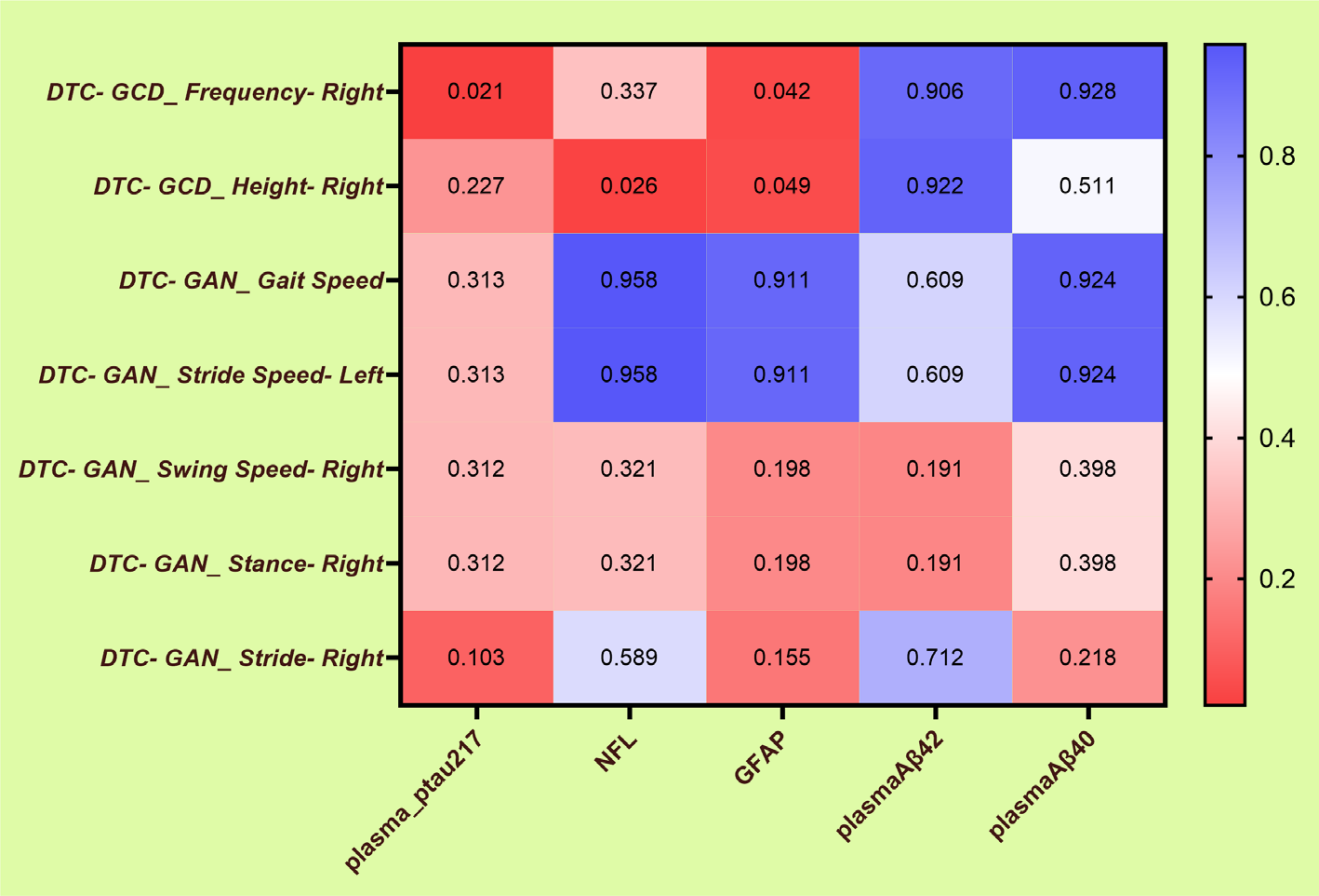
Heatmap of P values for associations between plasma biomarkers and dual‐task gait parameters in MCI+ patients. Heatmap showing P values for correlations between plasma biomarkers and gait parameters identified as independently associated with MCI+ status. Nominally significant associations were observed for DTC‐GCD_Frequency‐Right with p‐tau217 and GFAP, and for DTC‐GCD_Height‐Right with NfL and GFAP. Correlation coefficients are reported in the main text. p‐tau217, phosphorylated tau at threonine 217; NfL, neurofilament light chain; GFAP, glial fibrillary acidic protein.

We further examined the correlations between plasma biomarkers and the gait parameters identified as independently associated with MCI+ status. In MCI+ patients, plasma p‐tau217 was negatively correlated with DTC‐GCD_Frequency‐Right (r = −0.401, *p* = 0.021), plasma NfL was negatively correlated with DTC‐GCD_Height‐Right (r = −0.394, *p* = 0.026), and plasma GFAP was negatively correlated with both DTC‐GCD_Frequency‐Right (r = −0.345, *p* = 0.042) and DTC‐GCD_Height‐Right (r = −0.351, *p* = 0.049). These correlations were further evaluated with FDR correction for multiple comparisons (Figure 1).

### 3.6 The Diagnostic Value of Gait Analysis and Plasma Biomarkers Combined With Gait Analysis for MCI+ Patients

**FIGURE 2:**
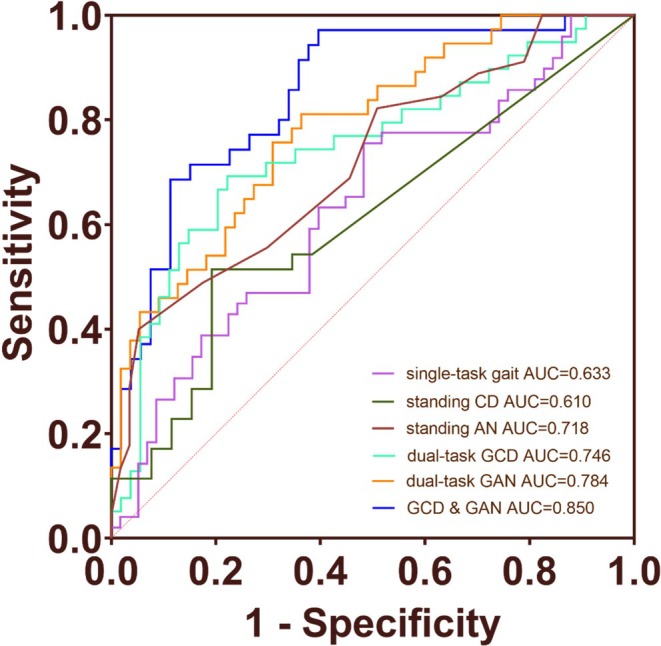
Diagnostic value of gait analysis and cognitive tasks for MCI+ from CN. ROC curve analysis was conducted to evaluate the ability of gait analysis and cognitive tasks to identify MCI+ from CN. The combination of GCD and GAN had the highest AUC. AUC, area under the receiver operating characteristic curve; CD, countdown; AN, animal naming; GCD, gait‐countdown; GAN, gait‐animal naming.

**FIGURE 3:**
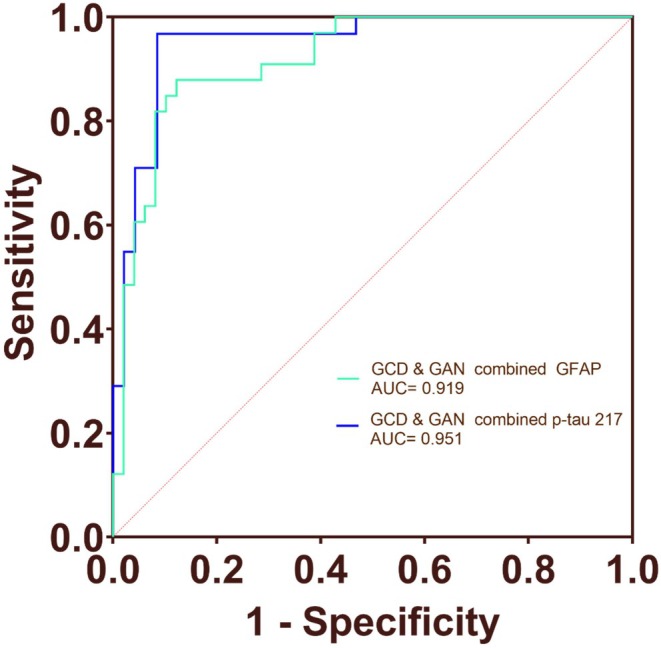
Diagnostic value of gait analysis and plasma biomarkers for MCI+ from CN. ROC curve analysis was conducted to evaluate the ability of gait analysis and plasma biomarkers to identify MCI+ from CN. The combined ROC curve of GCD, GAN, and plasma GFAP had an AUC value of 0.919 (*p* < 0.001). The combined ROC curve of GCD, GAN, and plasma p‐tau 217 had an AUC value of 0.951 (*p* < 0.001). AUC, area under the receiver operating characteristic curve; GCD, gait‐ countdown; GAN, gait‐ animal naming; p‐tau217, phosphorylated tau 217; NfL, neurofilament light chain; GFAP, glial fibrillary acidic protein.

To evaluate the ability of gait analysis, cognitive tasks, and plasma biomarkers combined with gait analysis to identify MCI due to AD patients from CN individuals, we conducted an ROC curve analysis. The results showed that the AUC value for single‐task gait analysis was 0.633 (*p* = 0.018), the AUC value for GCD was 0.746 (*p* < 0.001), and the AUC value for GAN was 0.784 (*p* < 0.001). The combined ROC curve of GCD and GAN had an AUC value of 0.850 (*p* < 0.001). The AUC value of standing CD (SCD) and standing AN (SAN) were 0.610 (*p* = 0.145) and 0.718 (*p* = 0.0002), respectively (Figure 2). The AUC values for plasma p‐tau 217, NFL, and GFAP were 0.936, 0.819, and 0.839, respectively (Figure S1). The combined ROC curve of GCD, GAN, and plasma GFAP had an AUC value of 0.919 (*p* < 0.001). The combined ROC curve of GCD, GAN, and plasma p‐tau 217 had an AUC value of 0.951 (*p* < 0.001) (Figure 3). Compared with the corresponding biomarker‐only models, the combined models showed numerically higher AUCs; however, these increases were not statistically significant in DeLong testing for the MCI+ vs. CN comparison.

### 3.7 The Value of Gait Analysis and Plasma Biomarkers Combined With Gait Analysis in MCI+ and MCI‐ Patients

**FIGURE 4:**
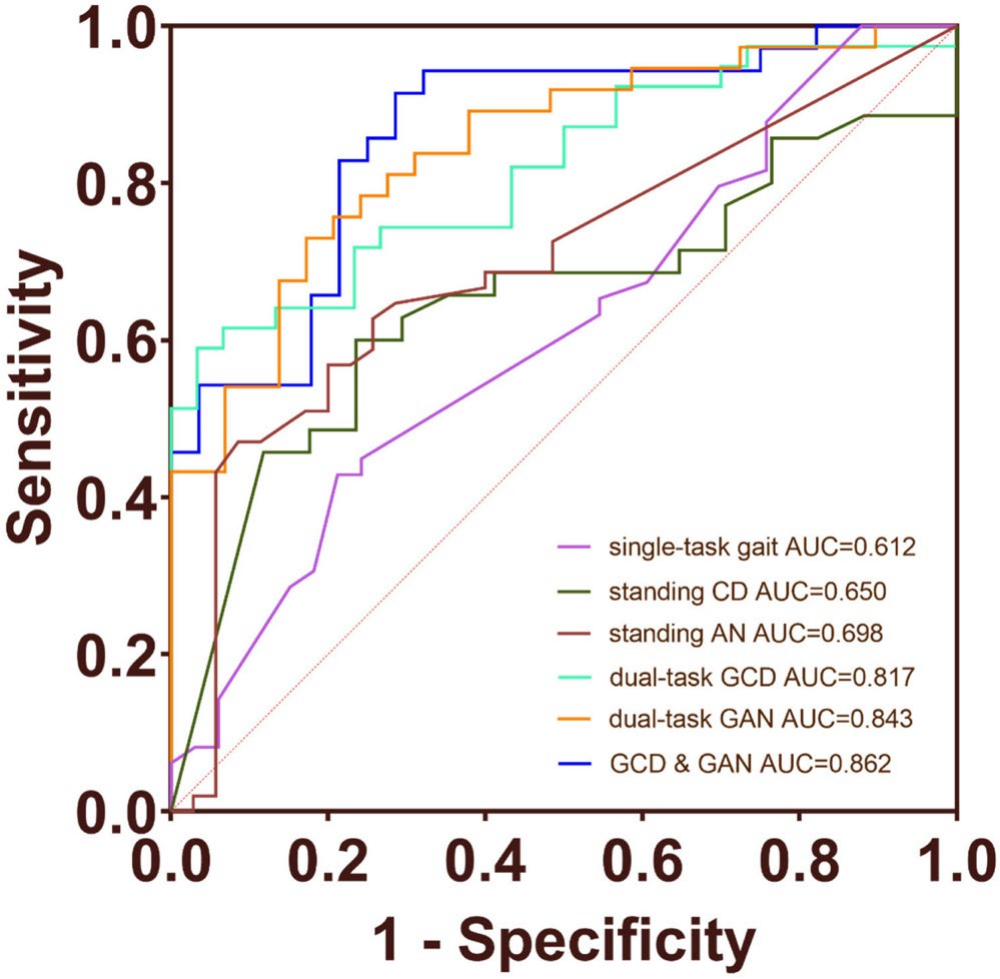
Diagnostic value of gait analysis and cognitive tasks for MCI+ from MCI‐. ROC curve analysis of gait analysis and cognitive tasks to discriminate MCI+ from MCI‐. The combination of GCD and GAN achieved the highest AUC value of 0.862. AUC, area under the receiver operating characteristic curve; CD, count down; AN, animal naming; GCD, gait‐countdown; GAN, gait‐animal naming.

**FIGURE 5:**
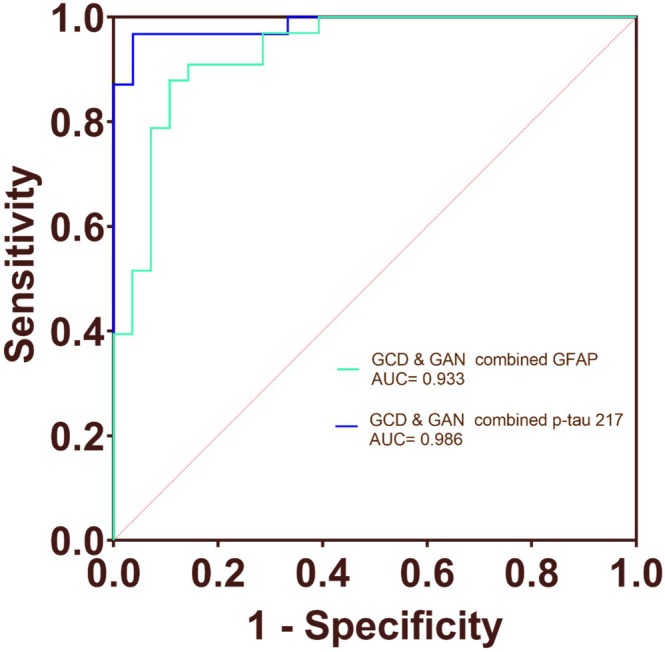
Diagnostic value of gait analysis and plasma biomarkers for MCI+ from MCI‐. ROC curve analysis was conducted to evaluate the ability of gait analysis and plasma biomarkers to identify MCI+ from MCI‐. The combined ROC curve of GCD, GAN, and plasma GFAP had an AUC value of 0.933 (*p* < 0.001). The combined ROC curve of GCD, GAN, and plasma p‐tau 217 had an AUC value of 0.986 (*p* < 0.001). AUC, area under the receiver operating characteristic curve; GCD, gait‐ countdown; GAN, gait‐ animal naming; p‐tau217, phosphorylated tau 217; NFL, neurofilament light chain; GFAP, glial fibrillary acidic protein.

In addition, to evaluate the predictive capability of gait analysis and cognitive tasks for MCI+ and MCI‐ patients, we conducted ROC curve analysis. The results showed that the AUC of single‐task gait was 0.612 (*p* = 0.087). The ROC curve of dual‐task gait analysis showed that the AUC of GCD was 0.817 (*p* < 0.001), and the AUC of GAN was 0.843 (*p* < 0.001). The ROC curve of combined GAN and GCD was further evaluated, with AUC = 0.862 (*p* < 0.001). The AUC values of SCD and SAN were 0.650 (*p* = 0.083) and 0.698 (*p* = 0.002), respectively (Figure 4). The AUC values for plasma p‐tau 217, NFL, and GFAP were 0.967, 0.614, and 0.755, respectively (Figure S2). The combined ROC curve of GCD, GAN, and plasma GFAP had an AUC value of 0.933 (*p* < 0.001). The combined ROC curve of GCD, GAN, and plasma p‐tau 217 had an AUC value of 0.986 (*p* < 0.001) (Figure 5). In DeLong testing, the improvement of the combined model over the GFAP‐only model was statistically significant, whereas the difference between the combined model and the p‐tau217‐only model was not statistically significant.

## 4 Discussion

In this prospective cohort study, participants were categorized into three distinct groups: MCI+, MCI‐, and CN groups. Consistent with previous research, MCI+ patients exhibited worse cognitive function than the MCI‐ group. Additionally, the MCI+ group had a higher proportion of APOE‐ε4 carriers, elevated plasma levels of p‐tau 217 and GFAP, and lower Aβ42 levels, further underscoring the distinct pathophysiological profiles of these subgroups.

The accurate identification of MCI+ is crucial as it enables the timely initiation of targeted medical interventions, such as anti‐amyloid therapies, which can potentially delay or mitigate cognitive decline [13, 36]. Amyloid PET imaging has emerged as an important tool for predicting future cognitive changes in patients with MCI, especially in clinical trials and treatment evaluations [13]. However, its widespread application is hindered by high costs, limiting its use in large‐scale screening. Traditional neuropsychological tests and clinical assessments, while capable of detecting subtle cognitive decline, often fall short in precisely determining the underlying etiology of MCI [37]. Gait analysis has emerged as a promising alternative for the early identification of AD and MCI patients. Nevertheless, many previous studies suffered from small sample sizes, relied solely on clinical diagnoses of MCI without pathological confirmation, and were constrained by outdated gait assessment technologies, which limited their reliability and generalizability. Moreover, a significant gap exists in the literature regarding gait analysis, specifically in MCI+ and MCI‐ patients.

Our study revealed that dual‐task gait analysis accentuated gait abnormalities compared to single‐task gait analysis. Dual‐task GCD and GAN measurements were significantly related to cognitive functions, including executive function and attention processes. The DTC, which quantifies the impact of additional cognitive load on gait, was notably higher in MCI+ patients, indicating greater difficulty in coordinating cognitive and motor tasks simultaneously. After adjusting for potential confounders, significant differences in DTC between the MCI+ and MCI‐ groups persisted for dual‐task GCD and GAN measurements. These findings suggest that dual‐task gait parameters are more sensitive indicators of cognitive impairment than single‐task gait parameters. Consequently, dual‐task gait analysis is more sensitive in detecting gait abnormalities and cognitive function changes in MCI+ patients. The more pronounced gait abnormalities in MCI+ patients further imply a relationship between amyloid burden and motor function.

In line with previous reports, our study demonstrated that gait analysis yielded higher AUC values than traditional neuropsychological assessments, such as MMSE and MoCA, when distinguishing MCI+ from MCI‐ [38, 39]. The results from single‐task and dual‐task gait analysis in MCI suggest that combining the ROC curves of both tasks improved the sensitivity and specificity of MCI detection. Moreover, the integration of dual‐task gait with plasma GFAP or plasma p‐tau 217 significantly improved the diagnostic performance, indicating the potential of this combined screening tool for identifying MCI+. Notably, while previous gait analysis studies in MCI patients primarily compared MCI with AD or healthy controls, our study is the first to use amyloid PET scans to systematically compare gait characteristics between MCI+ and MCI‐ patients.

Our findings further demonstrated that dual‐task gait analysis outperforms single‐task gait analysis and cognitive tasks in predicting MCI+ patients from CN individuals. This suggests that dual‐task gait analysis may serve as a more sensitive method for detecting early cognitive impairment in MCI+ patients. Although previous studies have reported that combining dual‐task gait with cognitive tasks (such as counting down) improved the discriminative ability between MCI and healthy controls [40, 41, 42], the AUC values obtained in our study were higher. This discrepancy may be attributed to the fact that our MCI patients were amyloid PET‐positive, whereas those in previous studies were diagnosed clinically without pathological confirmation. While the predictive values of our finding do not yet match those of PET or cerebrospinal fluid (CSF) biomarkers [43], they offer a more practical and time‐efficient approach for the early detection of MCI+ compared to neuropsychological tests. Moreover, the combination of dual‐task gait with plasma GFAP or p‐tau217 yielded higher AUCs than the corresponding biomarker‐only models, although not all between‐model differences reached statistical significance in DeLong testing.

Despite its promise, gait analysis for the early identification of AD and MCI patients is not without limitations. The lack of standardized methods and criteria across different studies has led to poor comparability of results. In past research, passive, accelerometer‐based, and wearable devices were commonly used for physical activity and gait measurement in population and clinical studies. Although these early studies were hampered by small sample sizes and outdated wearable sensor systems, technological advances have improved the accuracy of gait measurement. The ReadyGo system used in this study, which does not rely on wearable devices, provided objective and representative data.

Among the emerging plasma biomarkers for AD, including p‐tau217, p‐tau181, NFL, and GFAP, p‐tau217 has been established as the most AD‐specific biomarker, with superior discriminative validity between MCI+ and MCI [23, 24]. Our results showed that p‐tau217 has the highest sensitivity and specificity for diagnosing MCI+ patients, followed by GFAP. Mechanistically, GFAP, an astrocytic intermediate filament protein, reflects the secondary activation of astrocytes after neuronal damage [30]. Multiple cohort studies have also confirmed the correlation between plasma GFAP concentrations and cerebral amyloid deposition [44, 45]. Previous research has consistently demonstrated the close association of plasma GFAP, NFL, and p‐tau217 with disease progression [46]. Our study further revealed that these plasma biomarkers were correlated with dual‐task gait features in MCI+ patients, indicating that dual‐task gait analysis not only has diagnostic potential but also aids in monitoring disease progression.

This study has several limitations. Firstly, due to the limited sample size, we were unable to conduct further stratified analysis of MCI patients by gender and age. Future studies should explore the differences in gait analysis between male and female patients, as well as those with early‐onset and late‐onset MCI. Secondly, our investigation focused solely on the correlation between serological indicators and gait analysis in MCI patients, lacking an analysis of CSF markers. Subsequent studies should explore the possible correlation between gait analysis and CSF markers. Thirdly, the current study of gait analysis in MCI patients was cross‐sectional; longitudinal studies are needed in future follow‐up to fully evaluate the predictive ability of gait for the progression of MCI to AD.

## 5 Conclusion

Our study provides evidence that gait analysis is a viable method for differentiating MCI+ patients from MCI‐ and CN populations, with dual‐task gait analysis offering superior predictive power compared to single‐task gait analysis. The integration of gait parameters with plasma biomarkers may improve the diagnostic performance for detecting MCI+ patients, although the incremental value over biomarker‐only models requires further validation. Additionally, dual‐task gait analysis is associated with disease severity and can be a valuable tool for monitoring disease progression in MCI+ patients.

## Author Contributions

Jiong Shi, Zhaozhao Cheng, Yong Shen, and Jiaonan Wu designed this study. Jiaonan Wu, Zhaozhao Cheng, and Xinyi Lv recruited patients and collected samples. Jiaonan Wu, Fang Tang, Yiwei Wang, and Feng Gao performed the experiments and analyzed the data. Jiong Shi and Jiaonan Wu interpreted the data and wrote the manuscript.

## Funding

This work was supported by the Strategic Priority Research Program of the Chinese Academy of Sciences (XDB39000000), Anhui Provincial Key R&D Programs (202304295107020056), Fundamental Research Funds for the Central Universities (YD9100002033), Natural Science Research Projects in Anhui Province Universities (2023AH053410), Anhui Provincial Clinical Medical Research and Translation Special Fund (202304295107020051), and Natural Science Foundation of Anhui Province (2308085QH265).

## Ethics Statement

The protocol was approved by the First Affiliated Hospital of USTC's Ethics Committee (IRB#2019KY‐26, new revision supplement: 2023KY‐117).

## Consent

All participants in the present study and/or their legally authorized representatives provided signed informed consent.

## Conflicts of Interest

The authors declare no conflicts of interest.

## Supporting information


**Figure S1:** Diagnostic value of plasma biomarkers for MCI+ from CN.

**Figure S2:** Diagnostic value of plasma biomarkers for MCI+ from MCI‐.

**Table S1:** Single‐task gait features among MCI+, MCI‐, and CN groups.

**Table S2:** Dual‐task GAN among MCI+, MCI, and CN groups.

**Table S3:** DTC‐ GAN among MCI+, MCI, and CN groups.

## Data Availability

The data that supports the findings of this study are available in the (Figure S1 and Figure S2) and (Tables [Link], [Link], [Link]) of this article.
