# Supplementary figures and images for: Multimodal Integration of Gait Dysfunction, Amyloid PET, and Plasma Biomarkers for Differentiating Etiological Subtypes in Mild Cognitive Impairment
Source: CNS Neurosci Ther. 2026 Jun 5;32(6):e70949. doi: 10.1002/cns.70949 (PMC13239215; doi:10.1002/cns.70949)

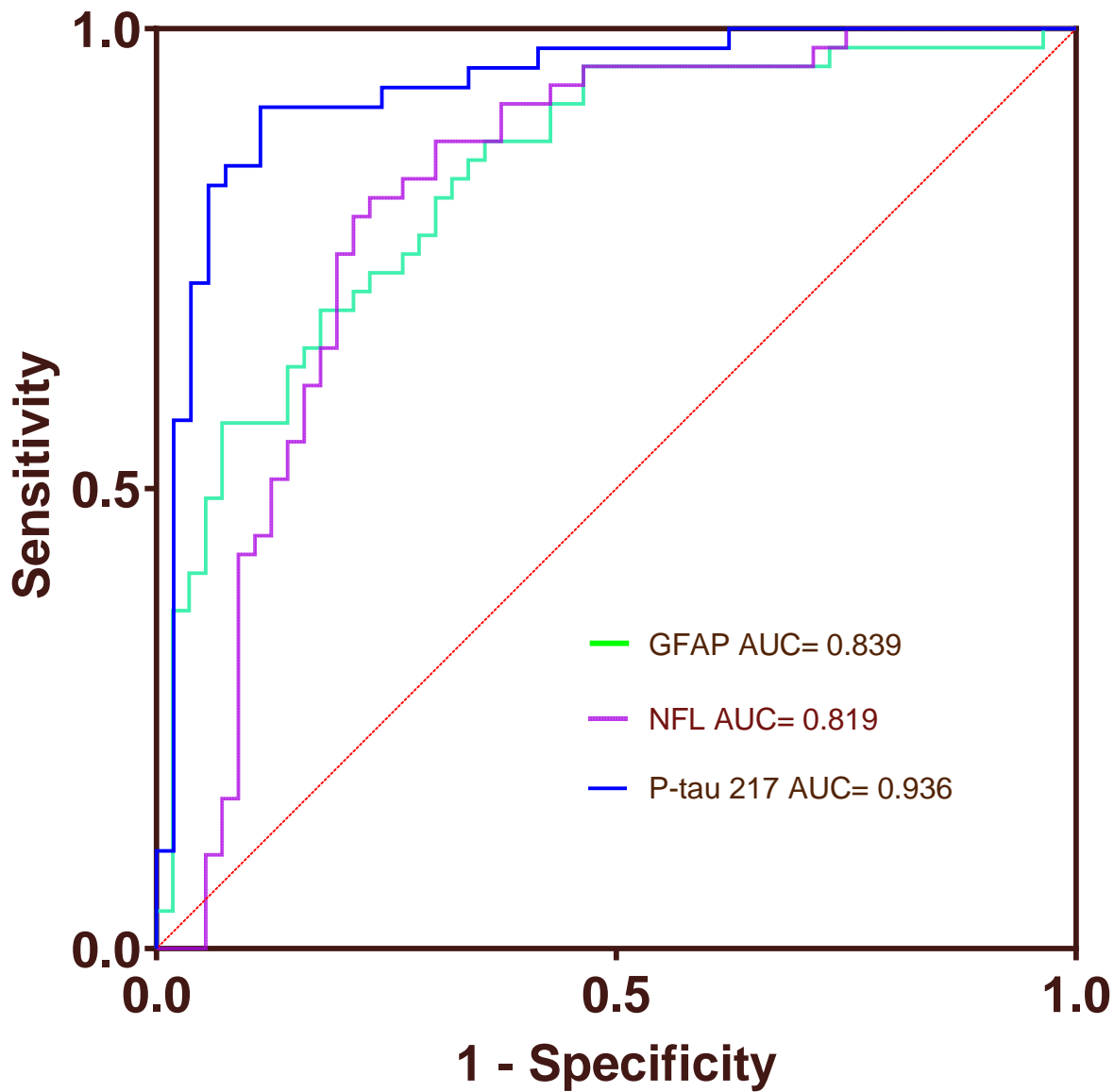

Supplement: Supplementary file 1 — Figure S1: Diagnostic value of plasma biomarkers for MCI+ from CN. [file CNS-32-e70949-s005.pdf]

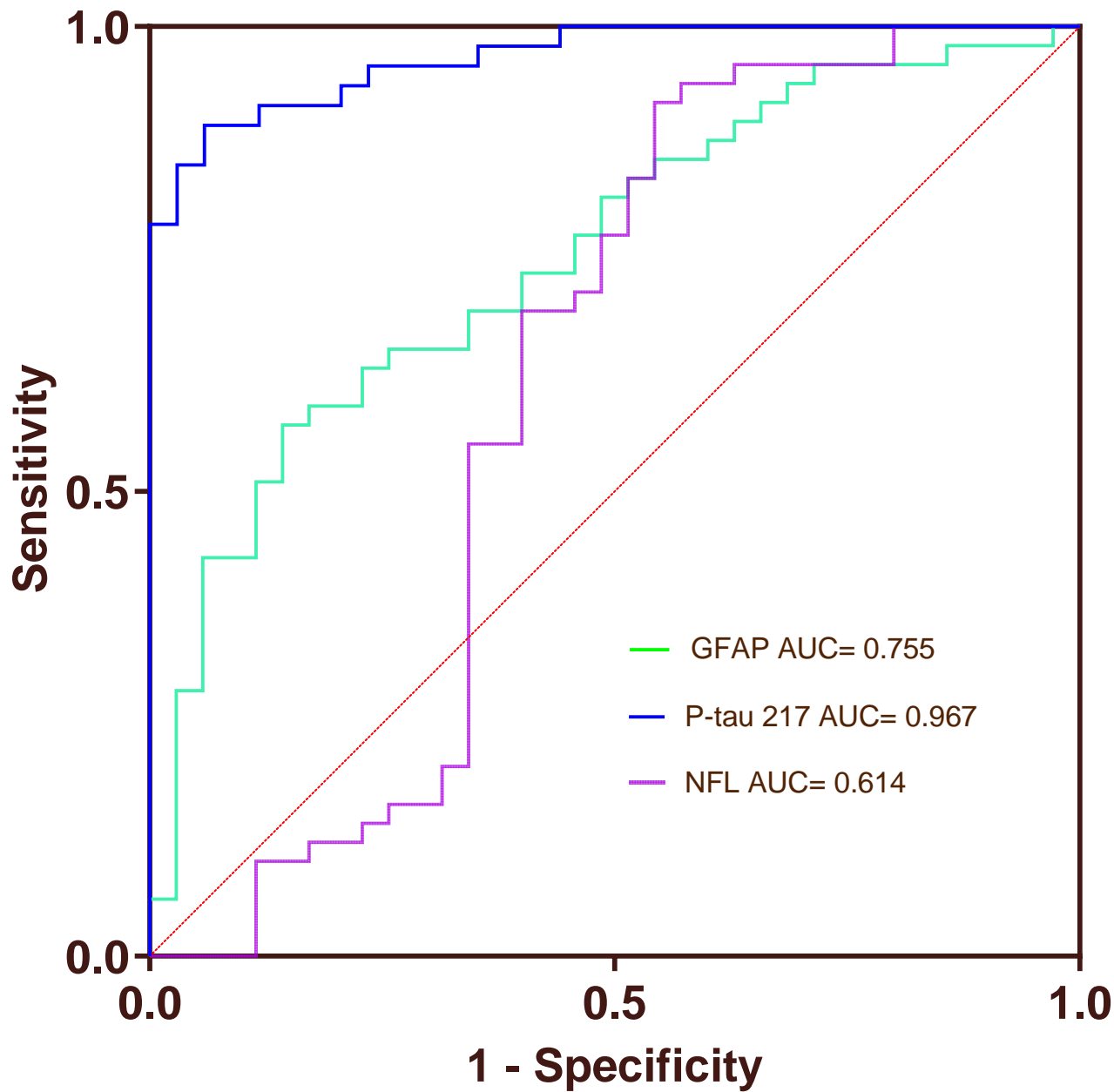

Supplement: Supplementary file 2 — Figure S2: Diagnostic value of plasma biomarkers for MCI+ from MCI‐. [file CNS-32-e70949-s002.pdf]
