# Supplementary material for: Multimodal Integration of Gait Dysfunction, Amyloid PET, and Plasma Biomarkers for Differentiating Etiological Subtypes in Mild Cognitive Impairment
Source: CNS Neurosci Ther. 2026 Jun 5;32(6):e70949. doi: 10.1002/cns.70949 (PMC13239215; doi:10.1002/cns.70949)
Supplement: Supplementary file 3 — Table S1: Single‐task gait features among MCI+, MCI‐, and CN groups. [file CNS-32-e70949-s004.docx]

Table S1 Single-task gait features among MCI+, MCI-, and CN groups

| Features | CN | MCI (+) | MCI (-) | P1 (CN vs. MCI+) | P2 (MCI- vs. MCI+) |
| --- | --- | --- | --- | --- | --- |
| Test Time | 41.85±9.39 | 46.61±13.90 | 43.98±9.42 | 0.036 | 0.339 |
| Standing Left | 67.50±1.25 | 67.82±1.73 | 67.40±2.50 | 0.278 | 0.37 |
| Standing Right | 67.46±1.93 | 67.52±1.62 | 67.49±1.04 | 0.865 | 0.912 |
| Swing Left | 32.49±1.25 | 32.17±1.73 | 32.59±2.51 | 0.277 | 0.37 |
| Swing Right | 32.53±1.93 | 32.47±1.62 | 32.50±1.04 | 0.866 | 0.91 |
| Bilateral Support Left | 35.05±2.09 | 35.59±2.63 | 35.07±2.53 | 0.242 | 0.379 |
| Bilateral Support Right | 35.16±2.67 | 35.35±2.57 | 35.33±2.15 | 0.706 | 0.963 |
| Stride Width | 0.13±0.02 | 0.13±0.02 | 0.12±0.02 | 0.782 | 0.333 |
| Stride Left | 1.20±0.19 | 1.15±0.16 | 1.13±0.14 | 0.193 | 0.586 |
| Stride Right | 1.20±0.18 | 1.15±0.17 | 1.15±0.15 | 0.131 | 0.95 |
| Height Left | 0.12±0.02 | 0.13±0.02 | 0.12±0.02 | 0.12 | 0.104 |
| Height Right | 0.11±0.02 | 0.12±0.02 | 0.11±0.02 | 0.135 | 0.165 |
| Speed | 1.02±0.23 | 0.97±0.16 | 1.01±0.18 | 0.206 | 0.311 |
| Frequency Left | 109.70±7.35 | 105.36±10.93 | 109.46±12.97 | 0.021 | 0.126 |
| Frequency Right | 108.88±11.72 | 104.43±11.42 | 109.89±12.23 | 0.05 | 0.042 |
| Stride Velocity Left | 1.10±0.19 | 1.00±0.15 | 1.04±0.20 | 0.004 | 0.335 |
| Stride Velocity Right | 1.10±0.18 | 1.03±0.16 | 1.06±0.19 | 0.006 | 0.196 |
| Swing Velocity Left | 2.53±0.41 | 2.40±0.34 | 2.45±0.42 | 0.068 | 0.602 |
| Swing Velocity Right | 2.59±0.38 | 2.42±0.34 | 2.46±0.42 | 0.019 | 0.607 |
| Turn Time | 1.69±0.53 | 1.94±0.73 | 1.97±0.61 | 0.044 | 0.833 |
| Coordination | -1.11±5.72 | -0.29±6.19 | -0.96±5.29 | 0.478 | 0.607 |
| Stride Time Variance Left | 6.72±6.77 | 6.22±4.10 | 5.35±3.10 | 0.644 | 0.299 |
| Stride Time Variance Right | 5.09±2.58 | 5.82±3.19 | 4.63±2.60 | 0.188 | 0.074 |
| Frequency Variance Left | 10.19±10.25 | 8.69±8.95 | 8.39±8.66 | 0.422 | 0.878 |
| Frequency Variance Right | 8.33±7.07 | 8.99±7.65 | 7.18±5.68 | 0.64 | 0.242 |

Abbreviations: CN, cognitively normal; MCI+, amyloid PET-positive mild cognitive impairment; MCI-, amyloid PET-negative mild cognitive impairment.
