# Supplementary material for: Multimodal Integration of Gait Dysfunction, Amyloid PET, and Plasma Biomarkers for Differentiating Etiological Subtypes in Mild Cognitive Impairment
Source: CNS Neurosci Ther. 2026 Jun 5;32(6):e70949. doi: 10.1002/cns.70949 (PMC13239215; doi:10.1002/cns.70949)
Supplement: Supplementary file 4 — Table S2: Dual‐task GAN among MCI+, MCI, and CN groups. [file CNS-32-e70949-s003.docx]

Table S2 Dual-task GAN among MCI+, MCI, and CN groups

| Features | CN | MCI(+) | MCI(-) | P1 (CN vs. MCI+) | P2 (MCI- vs. MCI+) |
| --- | --- | --- | --- | --- | --- |
| Test Time | 53.23±16.98 | 71.19±27.58 | 55.01±14.126.49 | ＜0.001 | 0.002 |
| Standing Left | 68.88±1.96 | 69.91±2.38 | 68.94±3.00 | 0.024 | 0.136 |
| Standing Right | 69.22±2.15 | 71.44±3.89 | 69.63±3.22 | 0.002 | 0.041 |
| Swing Left | 31.11±1.96 | 30.08±2.38 | 31.05±3.00 | 0.025 | 0.137 |
| Swing Right | 30.77±2.15 | 28.55±3.89 | 30.36±3.22 | 0.002 | 0.041 |
| Bilateral Support Left | 38.23±3.77 | 41.14±5.02 | 38.88±4.23 | 0.002 | 0.049 |
| Bilateral Support Right | 37.96±3.58 | 41.38±5.29 | 38.64±4.30 | ＜0.001 | 0.022 |
| Stride Width | 0.13±0.02 | 0.13±0.02 | 0.13±0.03 | 0.646 | 0.112 |
| Stride Left | 1.07±0.18 | 0.97±0.18 | 1.03±0.13 | 0.006 | 0.101 |
| Stride Right | 1.09±0.20 | 0.98±0.18 | 1.06±0.13 | 0.008 | 0.043 |
| Height Left | 0.11±0.02 | 0.11±0.02 | 0.11±0.02 | 0.857 | 0.839 |
| Height Right | 0.11±0.02 | 0.11±0.02 | 0.11±0.02 | 0.81 | 0.753 |
| Speed | 0.83±0.21 | 0.65±0.21 | 0.79±0.22 | ＜0.001 | 0.009 |
| Frequency Left | 95.38±13.54 | 85.58±16.32 | 93.91±21.57 | 0.002 | 0.08 |
| Frequency Right | 94.22±15.56 | 82.21±16.21 | 93.15±22.17 | ＜0.001 | 0.025 |
| Stride Velocity Left | 0.85±0.20 | 0.68±0.21 | 0.80±0.22 | ＜0.001 | 0.022 |
| Stride Velocity Right | 0.85±0.22 | 0.69±0.21 | 0.82±0.23 | ＜0.001 | 0.011 |
| Swing Velocity Left | 2.06±0.43 | 1.72±0.43 | 2.02±0.46 | ＜0.001 | 0.006 |
| Swing Velocity Right | 2.09±0.41 | 1.76±0.40 | 2.05±0.46 | ＜0.001 | 0.007 |
| Turn Time | 1.69±0.62 | 1.90±1.99 | 1.53±0.49 | 0.457 | 0.316 |
| Coordination | 1.22±6.51 | 4.79±10.44 | 2.05±6.53 | 0.058 | 0.205 |
| Stride Time Variance Left | 7.31±3.52 | 9.83±7.23 | 8.51±5.24 | 0.045 | 0.394 |
| Stride Time Variance Right | 8.62±9.83 | 10.35±7.95 | 7.66±4.49 | 0.356 | 0.096 |
| Frequency Variance Left | 10.55±8.58 | 10.70±8.92 | 13.99±23.37 | 0.936 | 0.411 |
| Frequency Variance Right | 9.27±6.36 | 11.35±9.33 | 10.72±8.31 | 0.196 | 0.77 |

Abbreviations: GAN, gait- animal naming; CN, cognitively normal; MCI+, amyloid PET-positive mild cognitive impairment; MCI-, amyloid PET-negative mild cognitive impairment.
