# Supplementary material for: Multimodal Integration of Gait Dysfunction, Amyloid PET, and Plasma Biomarkers for Differentiating Etiological Subtypes in Mild Cognitive Impairment
Source: CNS Neurosci Ther. 2026 Jun 5;32(6):e70949. doi: 10.1002/cns.70949 (PMC13239215; doi:10.1002/cns.70949)
Supplement: Supplementary file 5 — Table S3: DTC‐ GAN among MCI+, MCI, and CN groups. [file CNS-32-e70949-s001.docx]

Table S3 DTC- GAN among MCI+, MCI, and CN groups

| Features | CN | MCI+ | MCI- | P1 (CN vs. MCI+) | P2 (MCI- vs. MCI+) |
| --- | --- | --- | --- | --- | --- |
| Test Time | -0.29±0.30 | -0.62±0.53 | -0.32±0.33 | ＜0.001 | 0.004 |
| Standing Left | -0.02±0.03 | -0.03±0.04 | -0.02±0.07 | 0.085 | 0.459 |
| Standing Right | -0.03±0.04 | -0.06±0.05 | -0.03±0.05 | ＜0.001 | 0.017 |
| Swing Left | 0.04±0.06 | 0.07±0.08 | 0.04±0.10 | 0.092 | 0.227 |
| Swing Right | 0.05±0.10 | 0.13±0.10 | 0.07±0.09 | ＜0.001 | 0.041 |
| Bilateral Support Left | -0.09±0.10 | -0.17±0.13 | -0.12±0.14 | 0.004 | 0.127 |
| Bilateral Support Right | -0.09±0.10 | -0.19±0.14 | -0.09±0.11 | ＜0.001 | 0.004 |
| Stride Width | -0.06±0.13 | -0.08±0.16 | -0.02±0.09 | 0.489 | 0.054 |
| Stride Left | 0.12±0.09 | 0.17±0.10 | 0.10±0.09 | 0.018 | 0.01 |
| Stride Right | 0.11±0.10 | 0.16±0.11 | 0.09±0.07 | 0.029 | 0.003 |
| Height Left | 0.06±0.18 | 0.12±0.14 | 0.05±0.16 | 0.053 | 0.049 |
| Height Right | 0.08±0.17 | 0.13±0.18 | 0.05±0.13 | 0.168 | 0.051 |
| Speed | 0.18±0.25 | 0.35±0.17 | 0.23±0.17 | ＜0.001 | 0.005 |
| Frequency Left | 0.13±0.10 | 0.20±0.14 | 0.15±0.13 | 0.018 | 0.152 |
| Frequency Right | 0.15±0.11 | 0.22±0.13 | 0.16±0.15 | 0.002 | 0.059 |
| Stride Velocity Left | 0.24±0.14 | 0.33±0.17 | 0.24±0.15 | 0.005 | 0.024 |
| Stride Velocity Right | 0.24±0.14 | 0.33±0.16 | 0.22±0.16 | 0.005 | 0.008 |
| Swing Velocity Left | 0.21±0.13 | 0.30±0.15 | 0.18±0.17 | 0.004 | 0.004 |
| Swing Velocity Right | 0.20±0.11 | 0.28±0.13 | 0.18±0.14 | 0.003 | 0.002 |
| Turn Time | -0.08±0.50 | 0.03±0.84 | 0.16±0.23 | 0.458 | 0.4 |
| Coordination | 0.98±1.00 | 0.92±2.73 | 0.75±1.01 | 0.92 | 0.851 |
| Stride Time Variance Left | -0.48±0.90 | -0.86±1.11 | -0.76±1.17 | 0.072 | 0.721 |
| Stride Time Variance Right | -0.90±1.75 | -1.05±1.84 | -0.71±1.08 | 0.683 | 0.377 |
| Frequency Variance Left | -0.93±2.80 | -0.93±1.74 | -1.18±2.34 | 0.994 | 0.615 |
| Frequency Variance Right | -0.79±2.13 | -1.39±2.90 | -1.31±3.36 | 0.243 | 0.919 |

Abbreviations: DTC- GAN, Dual-task cost- gait- animal naming; CN, cognitively normal; MCI+, amyloid PET-positive mild cognitive impairment; MCI-, amyloid PET-negative mild cognitive impairment.
